# Correction to 29 articles due to inaccurate manuscript submission dates

**DOI:** 10.1093/ismejo/wraf008

**Published:** 2025-02-21

When The ISME Journal transitioned to its current manuscript submission system in September 2023, the original manuscript submission dates for content still undergoing peer review did not transfer properly. The affected article history dates and metadata have now been updated to reflect the accurate dates.

Affected papers:

• Xiaoqian Annie Yu, Craig McLean, Jan-Hendrik Hehemann, David Angeles-Albores, Fuqing Wu, Artur Muszynski, Christopher H Corzett, Parastoo Azadi, Elizabeth B Kujawinski, Eric J Alm, Martin F Polz, Low-level resource partitioning supports coexistence among functionally redundant bacteria during successional dynamics, *The ISME Journal*, Volume 18, Issue 1, January 2024, wrad013, https://doi.org/10.1093/ismejo/wrad013

• Yanchun Deng, Sa Yang, Li Zhang, Chenxiao Chen, Xuefen Cheng, Chunsheng Hou, Chronic bee paralysis virus exploits host antimicrobial peptides and alters gut microbiota composition to facilitate viral infection, *The ISME Journal*, Volume 18, Issue 1, January 2024, wrae051, https://doi.org/10.1093/ismejo/wrae051

• Cao Zheng, Dingqi Liu, Xinyu Lu, Huijun Wu, Jingyi Hua, Chuang Zhang, Kang Liu, Changchun Li, Jin He, Cuiying Du, *Trans*-aconitic acid assimilation system as a widespread bacterial mechanism for environmental adaptation, *The ISME Journal*, Volume 18, Issue 1, January 2024, wrae198, https://doi.org/10.1093/ismejo/wrae198

• Matthias Hoetzinger, Martin W Hahn, Linnéa Y Andersson, Nathaniel Buckley, Chelsea Ramsin, Moritz Buck, Julia K Nuy, Sarahi L Garcia, Fernando Puente-Sánchez, Stefan Bertilsson, Geographic population structure and distinct intra-population dynamics of globally abundant freshwater bacteria, *The ISME Journal*, Volume 18, Issue 1, January 2024, wrae113, https://doi.org/10.1093/ismejo/wrae113

• Hao Zhang, Ferdi L Hellweger, Haiwei Luo, Genome reduction occurred in early *Prochlorococcus* with an unusually low effective population size, *The ISME Journal*, Volume 18, Issue 1, January 2024, wrad035, https://doi.org/10.1093/ismejo/wrad035

• Etthel M Windels, Lloyd Cool, Eline Persy, Janne Swinnen, Paul Matthay, Bram Van den Bergh, Tom Wenseleers, Jan Michiels, Antibiotic dose and nutrient availability differentially drive the evolution of antibiotic resistance and persistence, *The ISME Journal*, Volume 18, Issue 1, January 2024, wrae070, https://doi.org/10.1093/ismejo/wrae070

• Branko Rihtman, Alberto Torcello-Requena, Alevtina Mikhaylina, Richard J Puxty, Martha R J Clokie, Andrew D Millard, David J Scanlan, Coordinated transcriptional response to environmental stress by a *Synechococcus* virus, *The ISME Journal*, Volume 18, Issue 1, January 2024, wrae032, https://doi.org/10.1093/ismejo/wrae032

• Mira D Liu, Yongle Du, Sara K Koupaei, Nicole R Kim, Monika S Fischer, Wenjun Zhang, Matthew F Traxler, Surface-active antibiotic production as a multifunctional adaptation for postfire microorganisms, *The ISME Journal*, Volume 18, Issue 1, January 2024, wrae022, https://doi.org/10.1093/ismejo/wrae022

• Wei Li, David Baliu-Rodriguez, Sanduni H Premathilaka, Sharmila I Thenuwara, Jeffrey A Kimbrel, Ty J Samo, Christina Ramon, Erik Anders Kiledal, Sara R Rivera, Jenan Kharbush, Dragan Isailovic, Peter K Weber, Gregory J Dick, Xavier Mayali, Microbiome processing of organic nitrogen input supports growth and cyanotoxin production of *Microcystis aeruginosa* cultures, *The ISME Journal*, Volume 18, Issue 1, January 2024, wrae082, https://doi.org/10.1093/ismejo/wrae082

• Camille Syska, Aurélie Kiers, Corinne Rancurel, Marc Bailly-Bechet, Justine Lipuma, Geneviève Alloing, Isabelle Garcia, Laurence Dupont, VapC10 toxin of the legume symbiont *Sinorhizobium meliloti* targets tRNASer and controls intracellular lifestyle, *The ISME Journal*, Volume 18, Issue 1, January 2024, wrae015, https://doi.org/10.1093/ismejo/wrae015

• Lingrui Qu, Chao Wang, Stefano Manzoni, Marina Dacal, Fernando T Maestre, Edith Bai, Stronger compensatory thermal adaptation of soil microbial respiration with higher substrate availability, *The ISME Journal*, Volume 18, Issue 1, January 2024, wrae025, https://doi.org/10.1093/ismejo/wrae025

• Sarah K Hu, Rika E Anderson, Maria G Pachiadaki, Virginia P Edgcomb, Margrethe H Serres, Sean P Sylva, Christopher R German, Jeffrey S Seewald, Susan Q Lang, Julie A Huber, Microbial eukaryotic predation pressure and biomass at deep-sea hydrothermal vents, *The ISME Journal*, Volume 18, Issue 1, January 2024, wrae004, https://doi.org/10.1093/ismejo/wrae004

• Corey Nelson, Pavani Dadi, Dhara D Shah, Ferran Garcia-Pichel, Spatial organization of a soil cyanobacterium and its cyanosphere through GABA/Glu signaling to optimize mutualistic nitrogen fixation, *The ISME Journal*, Volume 18, Issue 1, January 2024, wrad029, https://doi.org/10.1093/ismejo/wrad029

• Yohei Nishikawa, Ryota Wagatsuma, Yuko Tsukada, Lin Chia-ling, Rieka Chijiiwa, Masahito Hosokawa, Haruko Takeyama, Large-scale single-virus genomics uncovers hidden diversity of river water viruses and diversified gene profiles, *The ISME Journal*, Volume 18, Issue 1, January 2024, wrae124, https://doi.org/10.1093/ismejo/wrae124

• Xueyuan Du, Ning Liu, Bingfa Yan, Yisong Li, Minghao Liu, Ying Huang, Proximity-based defensive mutualism between *Streptomyces* and *Mesorhizobium* by sharing and sequestering iron, *The ISME Journal*, Volume 18, Issue 1, January 2024, wrad041, https://doi.org/10.1093/ismejo/wrad041

• Hong-Bin Liu, Hong-Xia Sun, Li-Qiong Du, Ling-Li Jiang, Lin-An Zhang, Yin-Yao Qi, Jun Cai, Feng Yu, Rice receptor kinase FLR7 regulates rhizosphere oxygen levels and enriches the dominant *Anaeromyxobacter* that improves submergence tolerance in rice, *The ISME Journal*, Volume 18, Issue 1, January 2024, wrae006, https://doi.org/10.1093/ismejo/wrae006

• Yue Zheng, Baozhan Wang, Ping Gao, Yiyan Yang, Bu Xu, Xiaoquan Su, Daliang Ning, Qing Tao, Qian Li, Feng Zhao, Dazhi Wang, Yao Zhang, Meng Li, Mari-K H Winkler, Anitra E Ingalls, Jizhong Zhou, Chuanlun Zhang, David A Stahl, Jiandong Jiang, Willm Martens-Habbena, Wei Qin, Novel order-level lineage of ammonia-oxidizing archaea widespread in marine and terrestrial environments, *The ISME Journal*, Volume 18, Issue 1, January 2024, wrad002, https://doi.org/10.1093/ismejo/wrad002

• Lauren E Manck, Tyler H Coale, Brandon M Stephens, Kiefer O Forsch, Lihini I Aluwihare, Christopher L Dupont, Andrew E Allen, Katherine A Barbeau, Iron limitation of heterotrophic bacteria in the California Current System tracks relative availability of organic carbon and iron, *The ISME Journal*, Volume 18, Issue 1, January 2024, wrae061, https://doi.org/10.1093/ismejo/wrae061

• Brandon M Stephens, Colleen A Durkin, Garrett Sharpe, Trang T H Nguyen, Justine Albers, Margaret L Estapa, Deborah K Steinberg, Naomi M Levine, Scott M Gifford, Craig A Carlson, Philip W Boyd, Alyson E Santoro, Direct observations of microbial community succession on sinking marine particles, *The ISME Journal*, Volume 18, Issue 1, January 2024, wrad010, https://doi.org/10.1093/ismejo/wrad010

• Moïra B Dion, Shiraz A Shah, Ling Deng, Jonathan Thorsen, Jakob Stokholm, Karen A Krogfelt, Susanne Schjørring, Philippe Horvath, Antoine Allard, Dennis S Nielsen, Marie-Agnès Petit, Sylvain Moineau, *Escherichia coli* CRISPR arrays from early life fecal samples preferentially target prophages, *The ISME Journal*, Volume 18, Issue 1, January 2024, wrae005, https://doi.org/10.1093/ismejo/wrae005

• Nan Yang, Henriette L Røder, Wisnu Adi Wicaksono, Birgit Wassermann, Jakob Russel, Xuanji Li, Joseph Nesme, Gabriele Berg, Søren J Sørensen, Mette Burmølle, Interspecific interactions facilitate keystone species in a multispecies biofilm that promotes plant growth, *The ISME Journal*, Volume 18, Issue 1, January 2024, wrae012, https://doi.org/10.1093/ismejo/wrae012

• Dikla Kolan, Esther Cattan-Tsaushu, Hagay Enav, Zohar Freiman, Nechama Malinsky-Rushansky, Shira Ninio, Sarit Avrani, Tradeoffs between phage resistance and nitrogen fixation drive the evolution of genes essential for cyanobacterial heterocyst functionality, *The ISME Journal*, Volume 18, Issue 1, January 2024, wrad008, https://doi.org/10.1093/ismejo/wrad008

• Junbeom Lee, Bohyun Jeong, Jeongtae Kim, Jae H Cho, Jin H Byeon, Bok L Lee, Jiyeun K Kim, Specialized digestive mechanism for an insect-bacterium gut symbiosis, *The ISME Journal*, Volume 18, Issue 1, January 2024, wrad021, https://doi.org/10.1093/ismejo/wrad021

• Paul Layoun, Mario López-Pérez, Jose M Haro-Moreno, Markus Haber, J Cameron Thrash, Michael W Henson, Vinicius Silva Kavagutti, Rohit Ghai, Michaela M Salcher, Flexible genomic island conservation across freshwater and marine *Methylophilaceae*, *The ISME Journal*, Volume 18, Issue 1, January 2024, wrad036, https://doi.org/10.1093/ismejo/wrad036

• Brittany N Zepernick, Emily E Chase, Elizabeth R Denison, Naomi E Gilbert, Alexander R Truchon, Thijs Frenken, William R Cody, Robbie M Martin, Justin D Chaffin, George S Bullerjahn, R Michael L McKay, Steven W Wilhelm, Declines in ice cover are accompanied by light limitation responses and community change in freshwater diatoms, *The ISME Journal*, Volume 18, Issue 1, January 2024, wrad015, https://doi.org/10.1093/ismejo/wrad015

• Yongchao Yin, Fadime Kara-Murdoch, Robert W Murdoch, Jun Yan, Gao Chen, Yongchao Xie, Yanchen Sun, Frank E Löffler, Nitrous oxide inhibition of methanogenesis represents an underappreciated greenhouse gas emission feedback, *The ISME Journal*, Volume 18, Issue 1, January 2024, wrae027, https://doi.org/10.1093/ismejo/wrae027

• Hui Lin, Donglin Wang, Qiaojuan Wang, Jie Mao, Yaohui Bai, Jiuhui Qu, Interspecific competition prevents the proliferation of social cheaters in an unstructured environment, *The ISME Journal*, Volume 18, Issue 1, January 2024, wrad038, https://doi.org/10.1093/ismejo/wrad038

• Biyan Huang, Yao Xiao, Yan Zhang, Asgard archaeal selenoproteome reveals a roadmap for the archaea-to-eukaryote transition of selenocysteine incorporation machinery, *The ISME Journal*, Volume 18, Issue 1, January 2024, wrae111, https://doi.org/10.1093/ismejo/wrae111

• Nobuto Takeuchi, Matthew S Fullmer, Danielle J Maddock, Anthony M Poole, The Constructive Black Queen hypothesis: new functions can evolve under conditions favouring gene loss, *The ISME Journal*, Volume 18, Issue 1, January 2024, wrae011, https://doi.org/10.1093/ismejo/wrae011

